# The Role of the TGFβ Receptor Signaling Pathway in Adult Beta Cell Proliferation

**DOI:** 10.3390/ijms19103136

**Published:** 2018-10-12

**Authors:** Yinan Jiang, Shane Fischbach, Xiangwei Xiao

**Affiliations:** 1 Division of Pediatric Surgery, Department of Surgery, Children′s Hospital of Pittsburgh, University of Pittsburgh School of Medicine, 4401 Penn Ave, Pittsburgh, PA 15224, USA; yinan.jiang12@chp.edu (Y.J.); shane_fischbach@brown.edu (S.F.); 2 The Warren Alpert Medical School of Brown University, 222 Richmond Street, Providence, RI 02903, USA

**Keywords:** diabetes, beta cell proliferation, Transforming growth factor β (TGFβ), SMAD7

## Abstract

Diabetes is a global epidemic and affects millions of individuals in the United States. Devising novel treatments for diabetes continues to be a great medical challenge. Postnatal beta cell growth or compensation is largely attributed to beta cell proliferation, which declines continuously with age. To boost beta cell proliferation to regenerate an adequate functional mass, there is a need to understand the signaling pathways that regulate beta cell proliferation for creating practical strategies to promote the process. Transforming growth factor β (TGFβ) belongs to a signaling superfamily that governs pancreatic development and the regeneration of beta cells after pancreatic diseases. TGFβ exerts its functions by activation of downstream Smad proteins and through its crosstalk with other pathways. Accumulating data demonstrate that the TGFβ receptor signaling pathway also participates in the control of beta cell proliferation. This review details the role of the TGFβ receptor signaling pathway in beta cell proliferation physiologically and in the pathogenesis of diabetes.

## 1. Introduction

Insulin is a key regulator of glucose homeostasis, and is produced exclusively by pancreatic beta cells. Inadequate secretion or action of insulin causes diabetes, a metabolic disease characterized by high blood glucose, persistence of which will lead to the classical symptoms of polyuria, polydipsia and polyphagia [1]. There are two major types of diabetes. While Type 1 diabetes (T1D) primarily results from the body’s failure to produce insulin due to autoimmunity to insulin-producing beta cells, Type 2 diabetes (T2D) primarily results from insulin resistance, a condition in which cells fail to use insulin properly; with time, these patients can develop an absolute insulin deficiency. In the United States, T1D and T2D affect approximately 30 million individuals [1]. The WHO estimates that there are about 347 million individuals who have diabetes across the globe. T1D and T2D are both associated with a deficiency of beta cells, although they are different diseases. Beta cell replication is viewed as the basic mechanism underlying beta cell generation and maintenance. However, human beta cell proliferation declines rapidly by 1 year of age [2,3]. Thus, diabetes patients will benefit from beta cell regeneration and replacement strategies.

**Figure 1:**
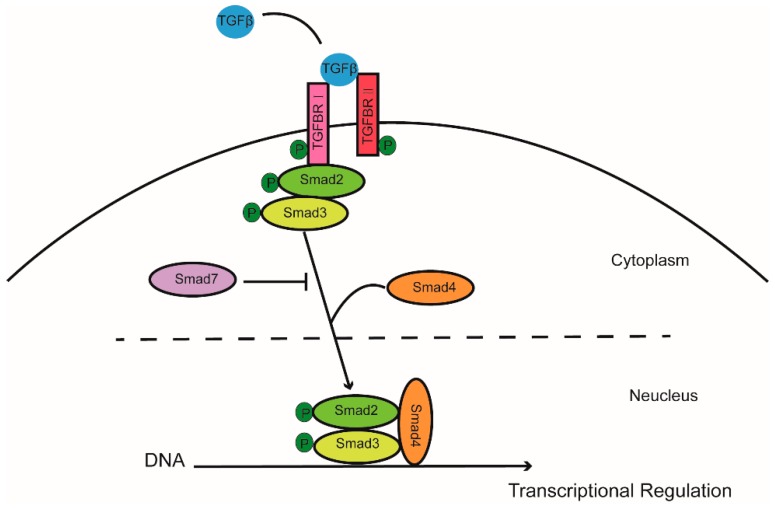
Canonical TGFβ/SMAD signaling pathway. TGFβ binds to type II receptors on the cell membrane, recruiting type I receptors to phosphorylate Smad2 and Smad3 (R-Smad) to form a complex with Smad4. The complex then translocates from the cytoplasm to the nucleus and regulate downstream gene expression.

Transforming growth factor β (TGFβ) belongs to a signaling superfamily which acts on plasma receptors to induce multiple biological effects, including development, immune response, and cell growth. In short, TGFβ binds to type II receptors on the cell membrane, recruiting type I receptors which then phosphorylate Smad2 and Smad3 (R-Smad). After forming a complex with Smad4, they translocate from the cytoplasm to the nucleus and regulate downstream gene expression. Activation of the TGFβ receptor signaling pathway may be inhibited by Smad6 and Smad7 [4] (Figure 1). TGFβ receptor signaling pathway plays roles in many cellular aspects through canonical SMAD pathway or noncanonical pathways, including mitogen-activated protein kinase (MAPK) pathways and the phosphatidylinositol 3′-kinase (PI3K)-protein kinase B (AKT) pathway. For example, Hamidi et al. showed that TGFβ activated PI3K in a tumor necrosis factor receptor-associated factor 6 (TRAF6)-dependent manner [5]. The TGFβ receptor signaling pathway has effects on beta cell proliferation and phenotype in mouse models. We have previously shown that Smad7, a potent suppressor of TGFβ receptor signaling, plays an essential role in maintenance of beta cell mass and in postnatal beta cell proliferation under certain circumstances [6,7,8]. In this review, we focus on the regulatory role of the TGFβ receptor signaling pathway in beta cell proliferation.

## 2. Beta Cell Proliferation and Cell Cycle Progression

The adult human pancreas contains about 1–2 g of beta cell mass, which plays an important role in glucose homeostasis [9]. During the embryonic period, human beta cells are primarily derived from precursor cells, after which proliferation of beta cells speeds up to generate functional beta cell mass. Significant increases in beta cell mass was observed to peak within the first 2 years of life, and then to rapidly decline in early childhood [10,11]. In the first year after birth, 1–3% of beta cells are in the active cell cycle, whereas almost 50% of cells from other organs could proliferate actively. In adults, human beta cell proliferation is very low, as no proliferation of beta cells was detected in 18 adult human pancreas samples based on staining for Ki-67, a specific cell proliferation marker that labels cells in an active cell cycle [12]. However, a recent study showed that the poor quality of Ki-67 staining in human tissue may result in underestimation of the real cell proliferation [13]. Hence, improvement of the technology or seeking alterative cell proliferation markers may be needed to determine and quantify beta cell growth in human. Although rodent beta cells exhibit better proliferation potential than human beta cells, generally, it is also very low [14].

Cell proliferation is first regulated at the G1/S cell phase, which controls the entry into the cell cycle. Given the low proliferation index of adult beta cells, it seems that cell cycle progression of beta cells is regulated by molecules which regulate G1/S transition [11,15,16]. Human adult beta cells contain not only cyclin-dependent kinases (CDKs) and transcription factors such as E2F that direct entry into the cell cycle, but also cyclin-dependent kinases inhibitors (CDKIs), as well as pocket proteins, including pRb, p107 and p130, which prevent cell cycle progression [17,18,19]. Replication of adult human beta cells cannot occur spontaneously or be easily stimulated by mitogen stimulation, largely due to the presence of CDKIs, which play a critical inhibitory role in cell cycle control. Overexpression of CDKs or cyclins in human islets transplanted into diabetic mice induced replication of adult beta cells and reverse diabetes effectively [20]. Blocking a CDKI, p57KIP2, by shRNA promoted entry into the G1/S phase. Inactivation of other CDKIs (such as p18INK4c, p21CIP1 and p27KIP1) induces familial syndromes of endocrine-cell hyperplasia [21,22]. In rodent beta cells, it was also observed that some cyclins, such as cyclinD2 and CDK6, were located in cytoplasm, rather than in nucleus in other cell types [19,23]. Similar result was reported in human beta cell [23]. Hence, the improper location of cyclins may also retrain beta cell proliferation in both rodents and humans. The proper balance of CDKs and CDKIs is likely coordinated to regulate the replication of adult beta cells.

## 3. TGFβ in Beta Cell Proliferation and Function

Past studies have shown that the TGFβ receptor signaling pathway participates in pancreatic development, as well as pancreatic disease like pancreatitis and pancreatic carcinoma [24,25,26,27]. TGFβ receptor signaling promotes endocrine cell differentiation and maturation and inhibits acinar cell growth during embryogenesis [28,29,30]. Our own observations have shown that beta cell proliferation occurs following pancreatic duct ligation (PDL), and that this proliferation depends on infiltrating macrophages [6]. Macrophages not only release TGFβ1 to upregulate Smad7, but also secrete Epidermal Growth Factor (EGF) to activate the EGF pathway, which inhibits the nuclear translocation of the phosphorylated SMAD complex. This inflammation-induced beta cell proliferation was associated with increased levels of Cyclin D1, Cyclin D2 and nuclear exclusion of p27 regulated by Smad7 [6]. TGFβ receptor signaling appears to be not essential for beta cell proliferation after partial pancreatectomy (PPx), an increased-workload-induced beta cell proliferation model [31], although inhibition of the TGFβ receptor signaling pathway may slightly increase beta cell proliferation and induce beta cell mass 1 week after PPx [32]. These results suggest that TGFβ receptor signaling is a central regulator of beta cell proliferation and homeostasis. Smad7, as an antagonist of the TGFβ receptor signaling pathway, appears to be a direct trigger of beta cell proliferation [6]. In adult islets, TGFβ1, Smad2 and Smad3 are expressed continuously while Smad7 is not detectable in insulin, glucagon and somatostatin cells. Smad7 expression re-emerged in cells after pancreas injury associated with increased beta cell proliferation [6,8]. Interestingly, inhibition of Smad7 expression in beta cells leads to a reduction in beta cell proliferation [8,33].

The TGFβ receptor signaling pathway also plays a key role in beta cell function. Totsuka Y. et al. showed that the insulin secretion increased by cultured islets which was incubated with TGFβ1, and this process was dependent on the activation of Smad2 [34,35,36]. Smad7 also participates in beta cell function and insulin secretion, since overexpression of Smad7 in beta cells results in reversible diabetes in mice [37]. Glucose concentration also influences insulin secretion induced by TGFβ1. TGFβ receptor signaling was shown to be crucial for insulin secretion in 200 mg/dL glucose concentrations without effects on beta cell proliferation, while TGFβ1 no longer induced insulin secretion when blood glucose reached 300 mg/dL [35,38]. These results suggest a central role for the TGFβ receptor signaling pathway in regulating beta cell function.

## 4. Crosstalk of TGFβ with Other Pathways in Beta Cell Proliferation

Inflammation occurs in both T1D and T2D and plays an important role in cell proliferation [39,40,41,42]. IL-1β, secreted by islets after hyperglycemic stimulation, participates in beta cell functional impairment and apoptosis [43]. IL-1β binds to its receptor (IL-1R) and triggers activation of the NF-κB (nuclear factor kappa-light-chain-enhancer of activated B cells) signaling pathway, which leads to FAS receptor upregulation and beta cell apoptosis [44,45]. Preventing expression of IL-1β could reverse diminished insulin expression and impaired beta cell function. Transgenic mice that express beta cell-specific NF-κB inhibitor demonstrate resistance to streptozotocin induced diabetes [46,47]. In summary, IL-1β and NF-κB mediate glucotoxic islet inflammation and beta cell apoptosis in diabetes.

TGFβ may also participate in glucotoxic islet inflammation and interacts with the NF-κB signaling pathway. Several studies have revealed that TGFβ family genes, including BMP5 and SMAD7, are upregulated by hyperglycemia. An abnormal reaction of TGFβ signaling leads to beta cell functional impairment and increased blood glucose [43,46,47,48]. TGFβ regulates keratinocyte differentiation and proliferation through interactions between smad2, smad3 and NF-κB submit IκB, kinase α (IKKα) [48]. Smad3 interacts with an NF-κB submit and regulates epithelial cell transcriptional activity and ECM related genes in fibroblasts [49]. It has also been reported that NF-κB suppresses the TGFβ signaling pathway by Smad7 activation. Together, crosstalk between NF-κB and TGFβ mediates numerous essential cell functions in multiple organs. However, the nature of interactions between TGFβ and NF-κB in beta cells remains to be studied.

It has also been reported that TGFβ and Insulin-like Growth Factor 1 Receptor (IGFR) signaling regulates the function of beta cells [50]. TGFβ and IGFR signaling are involved in cell proliferation through interaction with PI3K and R-Smads. FOXO family proteins are at the center of this process. Interaction between FOXO and Smad3/4 complex actives cell cycle inhibitor p21, which prevents neuroepithelial cell proliferation. Nucleus translocation and transcriptional function of FOXO could be inhibited by AKT, a downstream molecule of PI3K signaling pathway [51,52]. Negative regulation of FOXO by insulin receptor/IGFR signaling pathway has been shown to induce expression of Pdx1, MafA, the essential beta cell transcriptional factor [52]. Thus, FOXO appears to be a potential factor to regulate beta cell proliferation through coordinating the crosstalk between TGFβ and IGFR pathway.

It is well known that the EGF signaling pathway affects beta cell proliferation [53,54]. Global EGF receptor deficiency leads to a significant deficit in beta cell proliferation in the juvenile period, when the greatest proliferation of beta cells occurs. Similar results have been found in high fat diet and pregnancy mice. It was reported that TGFβ upregulates EGF expression via activation of MAPK and AKT [55]. There is also strong evidence for synergy between EGF and TGFβ signaling pathways in cancer cell proliferation [55]. Smad7 is upregulated by both TGFβ and EGF pathways [56]. Hence, similar synergy may occur in the beta cell proliferation process, and that Smad7 is the key regulator in this synergy. Given that the TGFβ receptor signaling pathway prevents beta cell proliferation, EGF signaling may suppress proliferation prevention induced by TGFβ though AKT and MAPK signaling pathways.

## 5. Proposed Mechanism for TGFβ-Regulated Beta Cell Proliferation during Pregnancy

Fetal growth depends on nutrition from the mother through the placenta during pregnancy [57]. Glucose provides the necessary energy and is transported across the placenta via a passive process. In the early stages of fetal development, glucose transport depends on a beta cell-induced gradient between mother and fetus [58]. In the later stages of pregnancy, this glucose concentration gradient is threatened by the developing fetus, which diverts increasing amounts of glucose from the mother [58]. To maintain the proper glucose gradient, the placenta secretes hormones to increase maternal blood glucose via insulin resistance and increased hepatic glucose production [59,60]. In rodents, beta cell mass expansion in pregnancy results from beta cell proliferation from existing beta cells [61]. Interestingly, the pregnancy-associated increases in beta cell mass parallels the rise of placental lactogens [62,63]. Placental lactogens and prolactin treatment drive rodent beta cell proliferation efficiently via ERK1/2 activity, and this process depends on the activation of beta cell prolactin receptor (PRLR), which is induced by placental lactogens and prolactin. Nicole et al. found that TGFβ1 inhibits PRL expression via downstream Smad activation [64]. These pioneering studies suggest a possible role of TGFβ receptor signaling in control of parental beta cell growth.

## 6. Discussion

Whereas the intestine relies on specialized stem cells to replenish and repair tissue cells, a stem cell appears to be absent in the pancreas [65]. Instead, pancreas depends primarily on mature cell types for cell regeneration under both homeostatic and injury conditions. The key challenge for beta cell replacement and regeneration is inducing beta cell replication via manipulating cell cycle molecules and underlying regulatory pathways. Several approaches, such as overexpression of CDKs and cyclins or inhibition of CDKIs (p18INK4c, p21CIP1 and p27KIP1), may increase beta cell replication. TGFβ receptor signaling pathway is well known for its function as a regulator of normal development. Emerging research has revealed that the TGFβ receptor signaling pathway also plays a central role in beta cell proliferation and functions, including glucose stimulated insulin secretion, compensatory beta cell insulin gene transcription. While beta cell proliferation in T2D may directly contribute to increases in functional beta cell mass, newly born beta cells in T1D are exposed to the autoimmune environment to be attacked. Our recent publication showed that reprogrammed beta cells from alpha cells by gene therapy may form new beta cells that are slightly different from normal beta cells, which spare them from immune attack for a certain period [66]. Hence, forming immune-innocent beta cells is exceptionally important in T1D. A recent concept of rendering differentiated beta cells back to a less differentiated or a stem-like state, a process called dedifferentiation, was found to be necessary for beta cell proliferation [43,67,68,69,70,71,72,73]. Accumulation of our knowledge of the complex network governing beta cell proliferation may eventually lead to successful beta cell replacement therapy.
